# Development and Validation of a Questionnaire to Assess Social Participation of High Risk-Adults in Germany During the COVID-19 Pandemic

**DOI:** 10.3389/fpubh.2022.831087

**Published:** 2022-04-26

**Authors:** Dominik Schröder, Gloria Heesen, Stephanie Heinemann, Eva Hummers, Alexandra Jablonka, Sandra Steffens, Marie Mikuteit, Jacqueline Niewolik, Tobias R. Overbeck, Jonathan Kallusky, Frank Müller

**Affiliations:** ^1^Department of General Practice, University Medical Center, Göttingen, Germany; ^2^Department of Rheumatology and Immunology, Hannover Medical School, Hanover, Germany; ^3^German Center for Infection Research (DZIF), Partner Site Hannover-Braunschweig, Hanover, Germany; ^4^Department of Hematology and Medical Oncology, University Medical Center Göttingen, Göttingen, Germany

**Keywords:** social participation, pandemic questionnaire, COVID-19, SARS-CoV-2, quality of life, questionnaire validation, questionnaire development and validation

## Abstract

**Background:**

Restrictions to contain the COVID-19 pandemic affect the social participation of people worldwide. Especially those at high risk for a severe disease tend to abstain from social gatherings. While there are a few questionnaires to measure social participation in elderly or chronic patients, a valid survey instrument that includes pandemic-related social participation is needed.

**Methods:**

We developed a social participation questionnaire that aims to assess pandemic-related restrictions in social participation. Items were developed using a theory and literature-based approach and then compiled in a discursive process involving experts and lay people. This was followed by the validation of the questionnaire through a cross-sectional survey on 431 individuals. Items with low item-total correlations and low factor loadings using exploratory factor analysis [EFA] were excluded. Using EFA on the remaining items, the factor structure was retrieved and tested with a confirmatory factor analysis [CFA]. Internal consistency was assessed with Chronbachs α.

**Results:**

Initially, 27 items were developed which were used for validation. 13 items were excluded due to low item-total correlations and factors loadings. EFA of the remaining 14 items revealed three factors which were identified as domains “active social participation,” “wellbeing,” and “restrictions”. CFA showed an acceptable model fit using the three-dimensional structure. Chronbachs α of 0.81 and McDonalds Ω of 0.87 indicate good internal consistency. Correlation analysis showed an association between the developed questionnaire and previously-established participation and mental health scales.

**Conclusion:**

This study suggests that our 14 item questionnaire is of high reliability and validity and can be used to measure social participation during a pandemic.

## Introduction

The still ongoing coronavirus disease [COVID-19] pandemic affects various aspects of life worldwide (1–6). Especially with dynamic changes of social restrictions, vaccine progress and occurrence of infection, the effect on how people pursue everyday life and participate in social activity of any kind can also change dramatically.

Until recently, social participation was discussed primarily in connection with people with physical, mental or sensory impairments of physiological functions, especially in the elderly (7, 8). The concept of social participation used in medical research has been adopted from the fields of geriatrics, disability research and rehabilitation (9–11). In these concepts, it is assumed that individual illnesses, symptoms or aging processes change or even limit an individual's ability to engage in social participation. Vice versa, social participation is generally associated with positive health outcomes. Improving social participation is one of the key strategies to combat the challenges of an aging population (12, 13). Known interventions to enhance social participation, in addition to medical and rehabilitation interventions, are to provide accessibility in various services like public transportation (14). As social participation can be summarized as “a person's involvement in activities that provide interaction with others in society or the community” and is thus a broad concept which also applies to pandemic situations and the impact of the restrictions on daily life during the course of a pandemic. Existing survey instruments often reflect the domains of the International Classification of Functioning, Disability and Health [ICF] or surrogates and are used to assess how specific individual medical conditions impact social participation but not a pandemic threat (15–17). Thus, these instruments do not address social fields affected by the pandemic (e.g. safety of the own person in the public space). Additionally, existing survey instruments are mainly used for rehabilitation research. Therefore, the need for new, validated, pandemic-appropriate instruments has become apparent. This is supported by the fact that especially so far non-validated, unstandardized or not fit-for-purpose instruments are being used in pandemic research (18–22).

Here, we describe the development and validation of a new questionnaire which was used to assess social participation during a pandemic in persons with a high-risk for a severe COVID-19 infection.

## Materials and Methods

The methods used in the development and validation of the questionnaire are based on current best practices (23, 24).

### Development of Items for the Pandemic Social Participation Questionnaire (PSP-Q)

The item development was performed in a discursive process following both deductive (literature review, assessment of existing scales) and inductive (group discussions on items with both experts and potential participants) approaches. Final refinement was undergone after pre-testing.

First, a theory- and literature-review using PubMed screening for articles on “social participation” and “quality of life questionnaire” in English and German language was undertaken. Additionally, we conducted a Google search for gray literature including national and international conventions and classification about social participation and rehabilitation. The purpose was to specify and identify domains and possible dimensions as well as assessment of existing scales.

The literature was fed back into a discursive process with authors and other experienced scientists from the Department of General Practice at University Medical Center Göttingen. We identified that existing questionnaires were based mainly on the International Classification of Functioning, Disability and Health [ICF] framework. The ICF, on the other hand, was considered hardly able to measure pandemic-specific impacts on social participation, as it assumes impairments to social participation only due to disease, as opposed to an external cause or hazard. Thus, emphasis was placed on identifying dimensions, that extend the existing framework of ICF. Following agreement in the group, we used the Annual Participation Report published by the German Federal Ministry of Labor and Social Affairs as the dimensional framework for social participation (25). This in turn is based in large parts on the United Nations Convention on the Rights of Persons with Disabilities (26). The framework includes the dimensions “Family and social network,” “Education and training,” “Employment and material life situation,” “Daily living,” “Health participation,” “Leisure, culture and sports,” “Security and protection of the own person,” and “Political and civil participation” and thus domains that were not recognized by ICF. Items were then derived interpreting existing survey instruments on social participation (27, 28), quality of life (29, 30), and the ICF (31) with the aim to provide at least two items per dimension. This resulted in a pool of items that were subsequently reduced by excluding duplicate items. It was consented not to pose questions but to provide statements on which probands can rate on a five-point Likert scale, whether they agree or disagree. Since certain items cannot be answered meaningfully in some circumstances (e.g. items concerning work life by retired persons), an additional category “not applicable to me” was added (32). The development of the items was based on the principle of comprehensibility; specifically, items should be formulated positively and negation should be avoided. Clear, simple sentence construction without abbreviations or technical terms was used. Particular attention was paid to statements about intensity, which ideally should be avoided. In total, 30 questions were derived from this first process. Questions were assigned in random order and compiled into a preliminary questionnaire.

Next, the first version of the questionnaire was discussed item by item in five sessions with each two people at high risk for a severe COVID course. This group was recruited pragmatically since the media reported the begin of the study before the first participant was included in the study. As a result, numerous people under immunosuppression came forward and expressed interest in participating in the study. Some of these individuals were approached and asked if they would be available for an open discourse about study questionnaires and their experiences during the COVID-19 pandemic. Group discussions were conducted as online video conferences in early spring 2021 when legal restrictions were in place on civil life. The group was given the task of speaking out loud about everything that comes to mind on each question and linking it to the participants' own current experiences, life situations, and expectations. As a result of this process, certain items were classified as too abstract (e.g., “I feel uncomfortable being close to others”) and transformed into more lifelike episodes based on participants' vivid experiences (“I hug friends and relatives to greet them when they are important and close to me”) (24). Additionally, the wording of the items was changed to be more precise and clear. In total, we developed 27 items during this phase. These items did not overlapped in every case with either ICF or the dimensional framework derived from the Convention on the Rights of Persons with Disabilities (26). However these items are needed to reflect the impact of the pandemic and we consented to use an *a posteriori* approach to identify domains.

For a pilot test of the PSP-Q, we asked 10 colleagues and their family members who are affiliated with the Department of General Practice but not involved in the questionnaire development to read and fill out the questionnaire. These persons were asked to provide feedback about the now article based questionnaire, regarding comprehensibility, and answerability, especially with regards to readability and layout. Additionally, we wanted to investigate how long it takes to complete the questionnaire. After this pilot test, the PSP-Q was finalized.

### Study Design and Participants

This questionnaire development and validation project is part of the CoCo Immune Study (33). In the CoCo Immune Study, participants with a high risk of a severe COVID-19 illness due to immunosuppressive therapy (e.g., due to autoimmune diseases or cancer treatment) or due to older age (participants aged 80+) were recruited for a 12-months observational study following COVID-19 vaccination. No intervention, treatment or counseling took place. Only participants aged 18 years or older were recruited.

We followed different recruitment strategies. To begin, potential participants were informed by local media reports, posters and flyers in private practices, vaccination centers, clinics and hospitals in the Southern Lower Saxony Region. Participants who contacted the study team and fulfilled the inclusion criteria were subsequently enrolled. Additionally, patients were enrolled who fit to the inclusion criteria and attended the outpatient clinics of the Department of Rheumatology and Immunology of the Hannover Medical School or the Department of Hematology and Medical Oncology of the University Medical Center Göttingen. Thus, recruitment was based on a pragmatic sample (real life sample).

### Data Collection and Management

At enrollment, participants completed a self-reported questionnaire on sociodemographic (age, gender, education level) and medical characteristics (diseases, pharmacotherapy), COVID-19 specific characteristics (previous SARS-CoV-2 infection, vaccine used for immunization) and the included scales. Data were entered into the EvaSys digital survey system (EvaSys GmbH, Lüneburg, Germany) and exported from there directly into SPSS data format. Only data from participants which completed all 27 items of the newly-developed questionnaire are used for statistical analyses.

### Measures

#### PSP-Q

The PSP-Q evaluates social participation with 27 items. A five-point likert-scale was used in all items ranging from 1 = strongly agree to 5 = strongly disagree. Additionally, participants had the possibility to state that question is not applicable to them which was then rated with the highest social participation as either strongly agree or strongly disagree depending on the poling of the item. To calculate the total score, negative items were reversed and summed up with all included items. Higher scores indicate a higher social participation with scores ranging between 27 and 135.

#### Patient Health Questionnaire-4 (PHQ-4)

The PHQ-4 is a brief, validated, high reliable (Cronbachs α 0.85) measure of anxiety and depression symptoms (34, 35). This scale consists of two subscales PHQ-2 for depressive symptoms and GAD-2 for anxiety, consisting of two four-point Likert-type items (0–3) for each subscale, and also produces an overall psychological distress sum score ranging from 0–12 while higher scores indicates impaired mental wellbeing. A sum score of ≥3 on either subscale or ≥6 on the whole scale is considered the cutoff point for identifying possible symptoms of clinical relevant anxiety or depression. Compared to the Brief Symptom Inventory, the PHQ-4 has a specificity of 94.5% and sensitivity of 51.6% (36).

#### Index for the Assessment of Health Impairments (IMET)

The IMET is a questionnaire to measure social participation based on the International Classification of Functioning, Disability and Health [ICF] (27, 28). It was initially developed to assess participation and involvement for persons suffering from a chronic disease. The main field of application is in the area of rehabilitation science research. The IMET is uni-dimensional and consists of 9 items with a 11 (0–10) level Likert-scale where higher scores indicate lower social participation consistently across all items. The sum of all 9 items can be used to determine the overall social participation with a high internal reliability (Cronbachs α 0.90). Higher scores indicate a lower level of social participation. The IMET was used during the COVID-19 pandemic by Mergel & Schützwohl to assess social participation before and after the lockdown in participants with a mental disorder and participants from the general population (22, 37).

In addition to the PHQ-4 and IMET, the health-related quality of life and subjective health status of the last 2 weeks was assessed each with a single item on a seven-point Likert-scale. Higher scores indicate a poorer health status or a lower quality of life.

### Data Analysis

Data analysis was conducted using two independent random samples stratified by gender. One sample was used for item analysis and exploratory-factor-analysis [EFA] (*n* = 215) to select items and extract factors. The other sample (*n* = 216) was used in confirmatory factor analysis [CFA] to verify the extracted factor structure from the EFA and assess the internal consistency on independent data.

#### Item Analysis and EFA

The individual items of the PSP-Q were examined using the mean, standard deviation, and the item-total correlation. Items with an item-total correlation of <0.30 were excluded from the final questionnaire.

The set of items were checked for eligibility to conduct an exploratory factor analysis using the KMO [Kaiser-Meyer-Olkin-Criteria] index score and Bartlett's test of sphericity (38, 39). A KMO index score of 0.8 or greater and a statistically significant Bartlett's test of sphericity indicate the eligibility of the items to conduct a principal component analysis [PCA]. The number of extracting factors was examined using parallel analysis (40). A PCA with varimax rotation was used to extract the factors and factor loading. Items were excluded with a factor loading below 0.4 or when a cross-loading between the primary and alternative factor loading with a distance ≤0.1 occurred. If an item was excluded the PCA was conducted again without the excluded items.

#### Construct Validity

To verify the extracted factor structure from the EFA, a confirmatory factor analysis [CFA] was conducted. Several indices were reported to assess the model fit. Reported indices were: Comparative Fit Index [CFI], Tucker Lewis Index [TLI], Root Mean Square Error of Approximation [RMSEA] and Standardized Root Mean Square Residual [SRMSR]. A close Model fit was determined by cut-off thresholds of 0.95 for CFI and TLI, 0.05 for RMSEAR and 0.06 for SRMR (41, 42).

Pearson correlations between the newly-developed questionnaire and already established questionnaires measuring similar constructs were calculated.Value thresholds of 0.1, 0.3, 0.5 stand for a small, medium, and large correlation, respectively (43).

#### Internal Consistency

Cronbachs α and McDonalds Ω was used to assess the internal consistency of the questionnaire and between individual factors extracted from the EFA. As for Chronbachs and McDonalds Ω values α ≥ 0.7 can be interpreted as acceptable, ≥ 0.8 as good and ≥ 0.9 as excellent (44, 45).

Further scores of the PSP-Q are tested with the Kolmogorov-Smirnov and Shapiro-Wilk test for a normal distribution. A non-significant result indicated a normal distribution of the data. The excess kurtosis and skewness will be additionally reported where values between −2 and +2 indicated a normal distribution of the data (46).

The statistical analysis was performed using the statistic software SPSS Version 27 (IBM, Armonk, NY) and R (Version 4.1.1). R was used to conduct and visualize the EFA, CFA and calculate Chronbachs alpha using the packages lavaan, lavaanPlots, paran and psych (47–50). If not stated otherwise, results were considered statistically significant if the *p* value was ≤ 0.05.

### Ethics

The study received approval by the Ethics Committee of the University Medical Center Göttingen (No. 29/3/21). All participants gave their written consent. The CoCo Immune Study is registered in the German Clinical Trials Register, an approved Primary Register in the WHO network (DRKS00023972).

## Results

**Figure 1:**
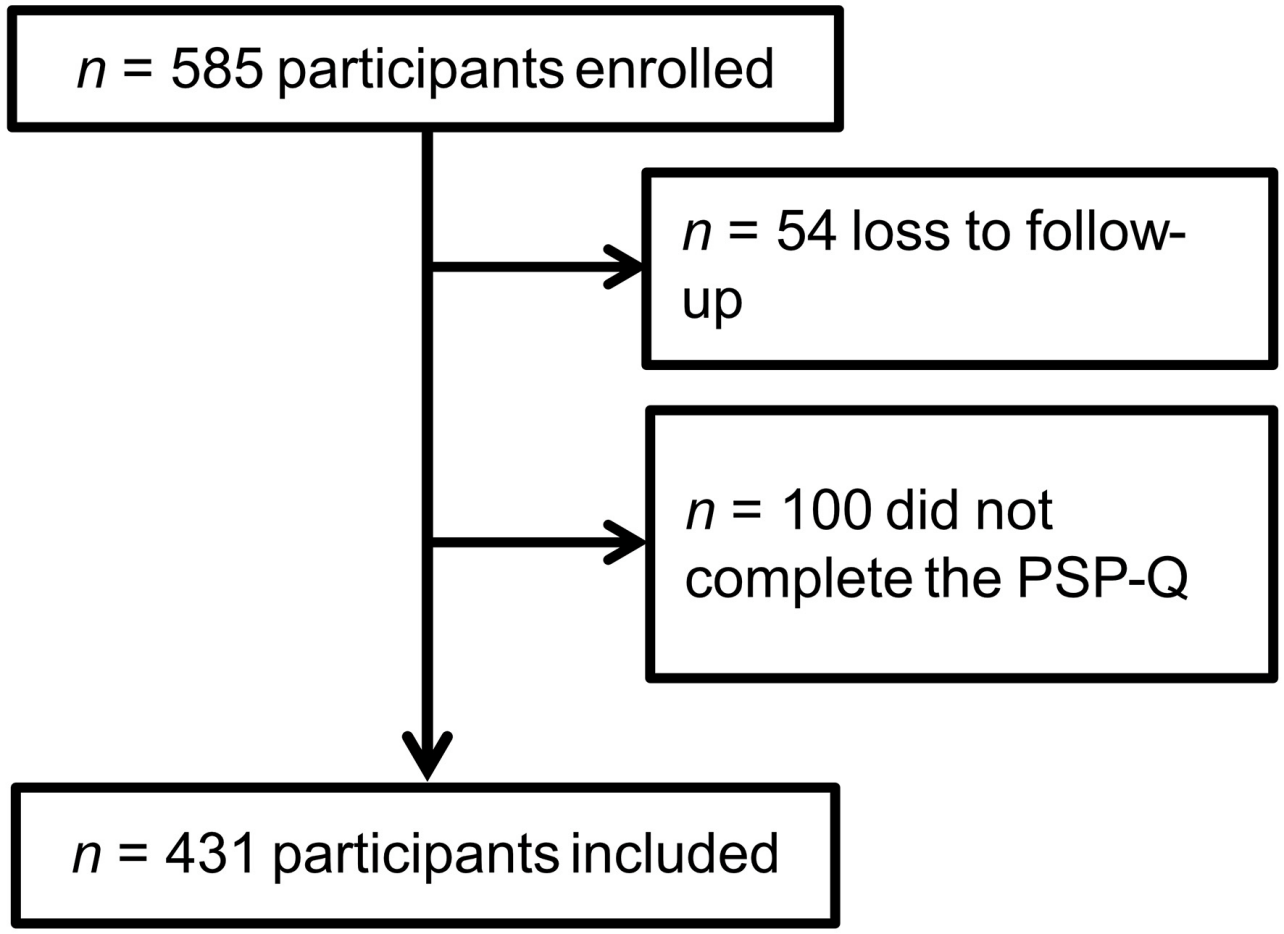
Flowchart of participants included in the analysis.

In total, 585 participants were enrolled in the study of which 54 were lost to follow-up (9.2%). Of these persons, 431 participants completed PSP-Q with all 27 items and this data was used for further statistical analysis (Figure 1). This data results in an item to participants' ratio of 1:15.9. The first participant completed the survey on March 30, 2021 and the last participant on September 2, 2021. The included participants were mostly female (57.7%). The ages ranged from 18 to 97 years with a mean age of 58.9 years. Nearly half of the participants (48.1%) had a college preparatory school education level (Table 1).

**Table 1:** Participants characteristics (*N* = 431).

**Gender**	
Female	241 (57.7)
Male	177 (42.3)
**Age, years**	
Mean (SD)	58,85 (16,52)
Median (IQR)	58 (23)
<40	60 (14.0)
40–65	210 (49.0)
>65	159 (37.1)
**School education^a^**	
Low	80 (19.2)
Middle	124 (29.8)
High	200 (48.1)
Other	12 (2.9)
Household^*^	
Parenting	74 (17.2)
Single parent	8 (1.9)
Living alone	105 (24.4)
Care of relatives	45 (10.4)
Morbidities^*^	
Hypertension	173 (40.1)
Heart failure	14 (3.2)
Diabetes type 2	31 (7.2)
Chronic obstructive pulmonary disease	14 (3.2)
Risk group^*^	
80+	57 (13.6)
Immunosuppressed	294 (70.3)
Active oncological treatment	94 (22.5)

*If not other stated data is n (%)*,

* *multiple selection possible*,

a *school education is based on secondary school level; SD, standard deviation; IQR, Interquartile range*.

**Figure 2:**
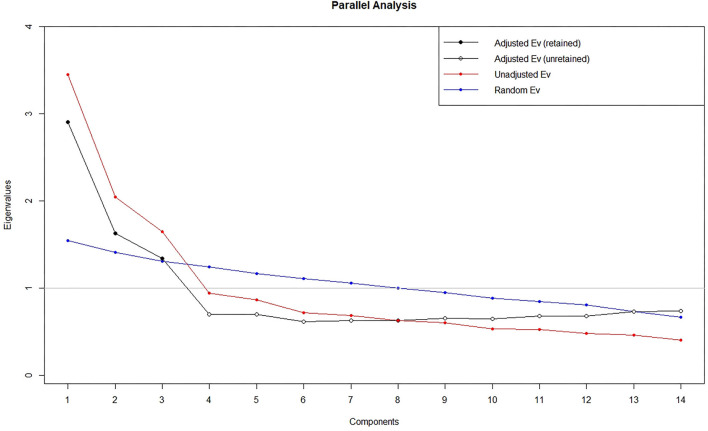
Parallel analysis scree plot.

Item-total correlations varied between 0.06 and 0.49, where eight items had an item-total correlation bellow 0.3 and were therefore excluded from further analysis. The remaining 19 items were eligible for an EFA with a Kaiser–Meyer–Olkin statistic of 0.82. The Bartlett's test of sphericity was also significant (x2(171) = 936, *p* < 0.01). Using parallel analysis adjusted eigenvalues ≤ 1 indicate three factors to extract in EFA. Using EFA four items had factor loadings <0.4 and one item had cross-loading with a distance ≤0.1. These five items were therefore excluded from further analysis. The EFA was recalculated without the excluded items and identified three latent constructs which explained 51.0% of the total variance (see Figure 2). The highest factor loadings on each item ranged from 0.48 to 0.74 (Table 2). The three extracted factors were interpreted by the researchers as domains of “wellbeing” (F1), “active social participation” (F2), and “restrictions” (F3).

**Table 2:** Factor loadings of the final items included in the PSP-Q.

	**Factor 1**	**Factor 2**	**Factor 3**
**% of Varaince explained**	**24.7**	**14.6**	**11.8**
Wellbeing 1	**0.52**		
Wellbeing 2	**0.48**		
Wellbeing 3	**0.55**		
Wellbeing 4	**0.57**		
Wellbeing 5	**0.52**		
Wellbeing 6	**0.61**		
Active social participation 1		**0.60**	
Active social participation 2		**0.74**	
Active social participation 3		**0.68**	
Active social participation 4		**0.56**	
Restrictions 1	0.36		**0.55**
Restrictions 2			**0.68**
Restrictions 3			**0.64**
Restrictions 4			**0.43**

*Factor loadings < 0.3 are omitted. Bold values indicate the assigned factor for each item*.

**Figure 3:**
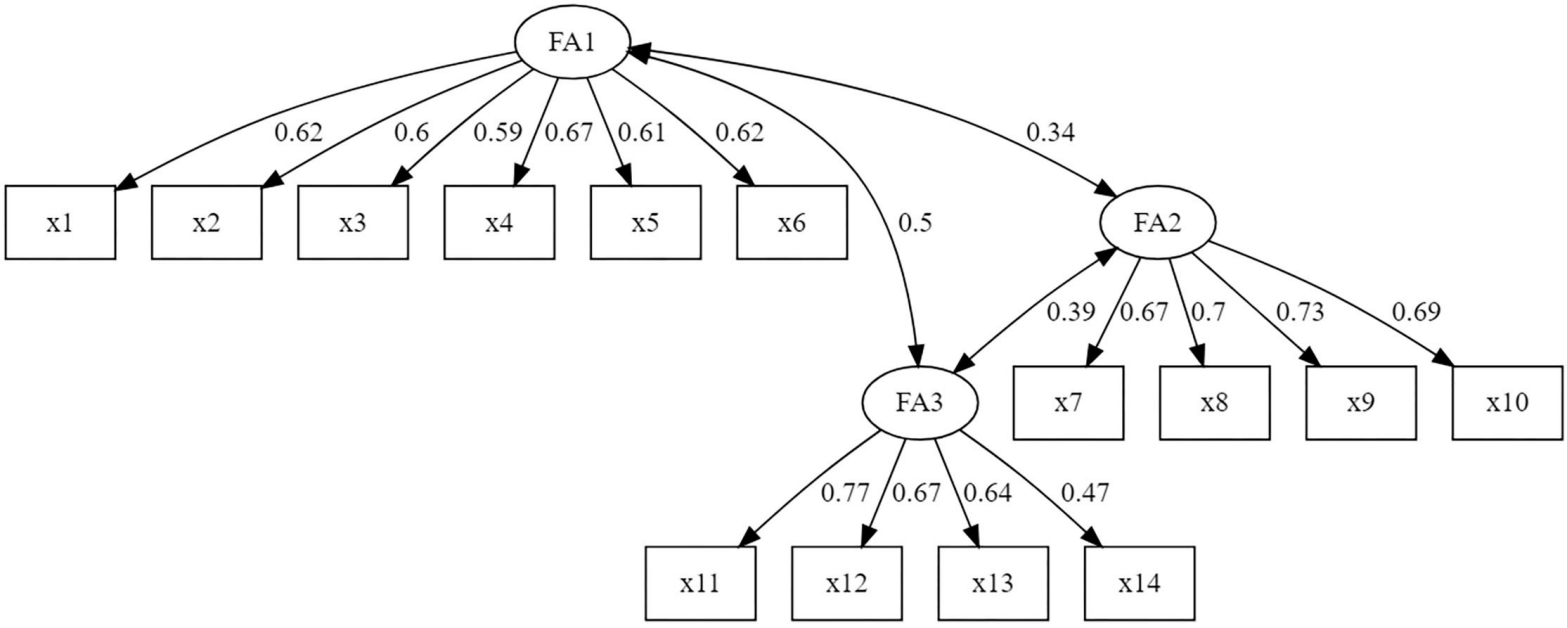
Three-factor model with standardized estimates.

The model fit indices of the three-factor model revealed by the EFA were: CFI = 0.94; TFI = 0.93; RMSEA = 0.054 (90% CI [0.036 – 0.070]) and SRMR 0.07 (Figure 3). Only RMSEA indicate a close model fit.

Significant negative correlations were found between the PSP-Q and all other included scales. A medium correlation could be found in the IMET, PHQ-4 and its sub-scales. Subjective health status indicates a small correlation and quality of life indicates a medium correlation with the PSP-Q. The second subscale interpreted as “active social participation” showed no significant correlation regarding the other included constructs (Table 3).

**Table 3:** Correlation between the PSP-Q and its subscales.

	**PSP-Q**	**PSP-F1**	**PSP-F2**	**PSP-F3**
IMET	**−0.34**	**−0.47**	**–**0.01	**−0.27**
**PHQ-4**	**−0.43**	**−0.58**	**–**0.05	**−0.30**
PHQ-2	**−0.41**	**−0.59**	**–**0.05	**−0.36**
GAD-2	**−0.36**	**−0.47**	**–**0.05	**−0.29**
Subjective health status	**−0.21**	**−0.27**	**–**0.03	**−0.24**
Quality of life	**−0.30**	**−0.42**	0.01	**−0.26**

*Bold indicates a significant association (p < 0.05); IMET, Index for the Assessment of Health Impairments; PHQ-4, Patient Health Questionnaire-4; PHQ-2, Patient Health Questionnaire-2; GAD-2, Generalized Anxiety Disorder Scale-2*.

To measure the internal consistency of the PSP-Q, Chronbachs α and McDonalds Ω was calculated. The PSP-Q as a whole had an α 0.81 where the α of the individual factors ranged from 0.70 to 0.78. McDonalds Ω was 0.84 for the whole scale and between 0.76 and 0.72 on the individual factors (Table 4).

**Table 4:** Chronbachs α of the PSP-Q and it subscales.

	**Chronbachs α (95% CI)**	**McDonalds Ω**
PSP-Q (14 items)	0.81 (0.78–0.85)	0.87
Factor 1 (6 items)	0.79 (0.74–0.83)	0.79
Factor 2 (4 items)	0.79 (0.74–0.83)	0.79
Factor 3 (4 items)	0.73 (0.67–0.79)	0.75

Sum scores of the PSP-Q ranged in the analyzed sample of 431 participants between 18 and 70 with a mean of 45.43 with a standard deviation of 10.64. Both, the Shapiro-Wilk test (p 0.58) and Kolmogorov-Smirnov test (p 0.13), yields a non-significant result which indicates a normal distribution of the questionnaire scores. Excessive kurtosis (−0.34) and skewness (−0.14) of the PSP-Q score distribution supports the Shapiro-Wilk and Kolmogorov-Smirnov test statistic with not crossing the cutoffs −2 or +2.

## Discussion

### Summary of Main Findings

The COVID-19 pandemic presents us with new challenges. Previous (social) participation questionnaires were developed for use in rehabilitation studies and these instruments focus on health impairments, social and work integration. With the COVID-19 pandemic, additional dimensions need to be addressed such as close social contact with family and friends and social restrictions. To add these pandemic-relevant aspects to existing dimensions of social participation, we developed the PSP-Q consisting of 14 items. Our results show that the PSP-Q is of high reliability and validity and can be used to measure social participation during a pandemic.

Social participation is a key construct reflecting a person's interactions with others and is associated with other constructs reflecting various health outcomes. Any medical treatment should aim to maintain or restore social participation. The recent COVID-19 pandemic and the social implications of the public health restrictions to decrease the spread of the SARS-CoV-2 virus have still not been fully explored. In particular, persons with at high risk for a severe COVID-19 disease course are challenged with complicated risk assessments about how much they should abstain from meeting others and engaging in social activities. Many uncertainties arise also regarding vaccines and vaccinations. Social participation can be a good concept to assess the impact of these challenges and uncertainties on behavior. The PSP-Q also expands the perspective about the impact of COVID-19 restrictions, measuring dimensions beyond the sphere of mental symptoms.

Already published studies measuring social participation in the COVID-19 pandemic have used newly-developed questionnaires or modified already existing scales that are not validated (19). Mergel and Schützwohl (22) used the IMET a participation scale developed to measure rehabilitation success to assess the effect of the pandemic lockdown in Germany on social participation (22). The PSP-Q could provide further insights regarding these research topics. Between the IMET and PSP-Q only a medium correlation was found. Further the subsscale “active social participation” shows no correlation with the IMET and other health-related measures. Our results show that the PSP-Q measures different aspects of social participation than the IMET and may reflect the social participation a pandemic more appropriate during. A comparison of these two measures in a longitudinal study evaluating different social restrictions during the pandemic is needed to reveal further differences between the two scales. Ammar et al. (19) found a negative impact of home confinement on social participation using a modified version of the Short Social Participation Questionnaire that was not validated (19). While the PSP-Q reflects the subjective agreement with a given statement the modified version of the Short Social Participation Questionnaire measures the actual social participation in a time frame.

The PSP-Q consists of 14 items which is on par with already existing multidimensional scales measuring participation (51–53). Further research should implement the PSP-Q in longitudinal studies to measure the influence of various population restriction measures and the effect of vaccination campaigns upon individual levels of social participation. One such policy example is the lifting of social restrictions in some countries (e.g., Denmark) with the COVID-19 pandemic still ongoing. Also, cultural differences need to be considered. In addition, the questionnaire was not exclusively designed for the current COVID-19 pandemic, but could also be used to measure social participation in other communicable diseases with pandemic or endemic dimensions. Possible implementation of the PSP-Q beyond the COVID-19 pandemic could include regional influenza epidemics. The PSP-Q is available in the Supplementary Material in German. An English translation of the questionnaire is included for reference, but this version was not used during the validation.

### Limitations

The development and validation of the questionnaire comes with limitations. Due to the pandemic situation and high-risk adults as the target group, the study was done with a minimium of personal contact and was therefore carried out in a more pragmatic way. For example, in-person focus group discussions with target or expert groups were not possible during the development of the questionnaire.

Over 25% of the participants of the initial 535 participants were excluded due to loss-to-follow-up or missing items in the PSP-Q. A loss-to-follow bias cannot be prevented. Also, missing answers could not be completely at random and therefore biased. The items of the questionnaires are to date only available in the German language. Persons with a high risk for severe COVID-19 infection in our sample were mostly taking immunosuppressive medication (70.3%). Only 13.6% of the sample were 80 years or older. Only high-risk adults were included which is why the use of the PSP-Q on a different target group needs re-validation.

The total explained variance by the three latent factors was 51.0%. Items with a factor loading below 0.4 on the highest loading factor were excluded. In the literature, this value differs between 0.3 and 0.5 with no clear consensus. As reliability criteria, only internal consistency was used in this analysis. The retest reliability was not feasible because social participation would differ between different time points during a pandemic e.g., with changing restrictions regarding social gatherings and cultural events. Only RMSEA met the criteria for a close model, where the other model fit indicies were close to the the cut off values and can be intereted as acceptable model fit. The choice of cut-off values of model fit indices varies in the literature with no clear consensus.

## Conclusion

The PSP-Q is a valid and reliable questionnaire with 14 items which assess social participation of high-risk groups during a pandemic. The sub-domains of the PSP-Q measure the dimensions “wellbeing,” “active social participation,” and “restrictions.” The strong correlation between the PHQ-4 and the sub-domain “wellbeing” of the PSP-Q showed an association between social participation and mental health. Nevertheless, the dimension “active social participation” showed no correlation with other questionnaires, indicating a missing dimension in the existing instruments. The PSP-Q can be used to measure the effect of various interventions and changes during the pandemic with regards to the effects upon social participation (e.g., social restrictions and vaccination progress) in high-risk groups.

## Data Availability Statement

The raw data supporting the conclusions of this article will be made available by the authors, without undue reservation.

## Ethics Statement

The studies involving human participants were reviewed and approved by Ethics Committee of the University Medical Center Göttingen. The patients/participants provided their written informed consent to participate in this study.

## Author Contributions

FM and AJ: research design. FM, GH, JN, TO, and JK: data collection. DS and FM: data analysis, interpreting results, and writing first draft. SS and SH: english-language editing. All authors writing and contributing to writing of the manuscript.

## Funding

The CoCo Immune Study is part of the DEFEAT-Corona Project funded by the European Regional Development Fund (ZW7-85152953).

## Conflict of Interest

The authors declare that the research was conducted in the absence of any commercial or financial relationships that could be construed as a potential conflict of interest.

## Publisher's Note

All claims expressed in this article are solely those of the authors and do not necessarily represent those of their affiliated organizations, or those of the publisher, the editors and the reviewers. Any product that may be evaluated in this article, or claim that may be made by its manufacturer, is not guaranteed or endorsed by the publisher.
