# Supplementary material for: Development and Validation of a Questionnaire to Assess Social Participation of High Risk-Adults in Germany During the COVID-19 Pandemic
Source: Front Public Health. 2022 Apr 26;10:831087. doi: 10.3389/fpubh.2022.831087 (PMC9086897; doi:10.3389/fpubh.2022.831087)
Supplement: Supplementary file 1 [file Table_1.pdf]

Supplementary Material

Was trifft auf Ihre **momentane** Lebenssituation zu?

		stimme voll zu	stimme eher zu	teils-teils	stimme eher nicht zu	stimme nicht zu	trifft auf mich nicht zu
1.1	**FA1:** Ich habe ausreichend Möglichkeiten, Aktivitäten außer Haus für meine körperliche und seelische Gesundheit (Sport, Spazieren gehen etc.) wahrzunehmen.	☐	☐	☐	☐	☐	☐
1.2	**FA1:** Ich fühle mich momentan häufig alleine.*	☐	☐	☐	☐	☐	☐
1.3	**FA1:** Ich habe momentan guten Kontakt zu all den Menschen, die mir wichtig sind.	☐	☐	☐	☐	☐	☐
1.4	**FA1:** Ich habe momentan ausreichend Betätigungen und Hobbies, die mir Spaß machen.	☐	☐	☐	☐	☐	☐
1.5	**FA1:** Momentan habe ich das Gefühl, das Leben läuft ohne mich weiter.*	☐	☐	☐	☐	☐	☐
1.6	**FA1:** Ich kann meinen Alltag weitgehend frei gestalten.	☐	☐	☐	☐	☐	☐
1.7	**FA2:** Ich umarme Freunde und Verwandte zur Begrüßung, wenn sie mir wichtig sind und nahe stehen.	☐	☐	☐	☐	☐	☐
1.8	**FA2:** Ich bin momentan aktiv in Vereinen, Kirchen, Selbsthilfegruppen oder der Nachbarschaft.	☐	☐	☐	☐	☐	☐
1.9	**FA2:** Ich habe schon für die nächsten Monate konkrete Zusammenkünfte, Urlaub oder Reisen geplant (bspw. ein Hotelzimmer/ Campingplatz reserviert oder einen Flug gebucht).	☐	☐	☐	☐	☐	☐
1.10	**FA2:** Ich entscheide mich spontan und ohne Planungen zu Verabredungen, zum Bummeln oder zu Ausflügen.	☐	☐	☐	☐	☐	☐
1.11	**FA3:** Ich schränke momentan mein Leben mehr ein als meine Freunde und Kollegen.*	☐	☐	☐	☐	☐	☐
1.12	**FA3:** Meine getroffenen Vorkehrungen, um mich vor einer Ansteckung mit COVID-19 zu schützen, sind strenger als die behördlichen Vorgaben (z.B. Anzahl der Menschen, die sich treffen dürfen).*	☐	☐	☐	☐	☐	☐
1.13	**FA3:** Persönliche Treffen mit für mich wichtigen Menschen außerhalb meines Haushalts vermeide ich.*	☐	☐	☐	☐	☐	☐
1.14	**FA3:** Ich verschiebe momentan Arzttermine, Krankenhausbehandlungen oder (Vorsorge-) Untersuchungen um mich nicht einer Infektionsgefahr auszusetzen.*	☐	☐	☐	☐	☐	☐

Supplementary Table 1. PSP-Q German; * indicates reversed pooled items; FA1: well-being participation; FA2: active social participation; FA3: restrictions

What applies to your **current** life situation?

	agree	rather agree	neither agree or disagree	rather disagree	disagree	does not apply to me
1.1 **FA2:** I have sufficient opportunities to engage in activities outside the home for my physical and mental health (Sports, walking, etc.).	☐	☐	☐	☐	☐	☐
1.2 **FA2:** I often feel alone at the moment.*	☐	☐	☐	☐	☐	☐
1.3 **FA2:** I am currently in good contact with all the people who are important to me.	☐	☐	☐	☐	☐	☐
1.4 **FA2:** At the moment I have enough activities and hobbies that I enjoy.	☐	☐	☐	☐	☐	☐
1.5 **FA2:** At the moment I feel like life goes on without me.*	☐	☐	☐	☐	☐	☐
1.6 **FA2:** I can organise my everyday life freely to a large extent.	☐	☐	☐	☐	☐	☐
1.7 **FA1:** I hug friends and relatives to greet them if they are important and close to me.	☐	☐	☐	☐	☐	☐
1.8 **FA1:** I am currently active in clubs, churches, self-help groups or the neighbourhood.	☐	☐	☐	☐	☐	☐
1.9 **FA1:** I have already planned specific meetings, holidays or trips for the next few months (e.g. reserved a hotel room/campsite or booked a flight).	☐	☐	☐	☐	☐	☐
1.10 **FA1:** I decide spontaneously and without planning to go on dates, strolls or excursions.	☐	☐	☐	☐	☐	☐
1.11 **FA3:** I am currently limiting my life more than my friends and colleagues.*	☐	☐	☐	☐	☐	☐
1.12 **FA3:** The precautions I have taken to protect myself from contracting COVID-19 are stricter than the official requirements (e.g. number of people allowed to meet).*	☐	☐	☐	☐	☐	☐
1.13 **FA3:** I avoid personal meetings with people outside my household who are important to me.*	☐	☐	☐	☐	☐	☐
1.14 **FA3:** I am currently postponing doctor's appointments, hospital treatment or (preventive) examinations in order not to expose myself to the risk of infection.*	☐	☐	☐	☐	☐	☐

Supplementary Table 1. PSP-Q German; * indicates reversed pooled items; FA1: wellbeing participation; FA2: active social participation; FA3: restrictions
